# Circular RNA microarray expression profile and potential function of circDOCK1 in colorectal cancer

**DOI:** 10.3389/fgene.2025.1443876

**Published:** 2025-02-04

**Authors:** Guojing Zhang, Xiaoyan Wu, Hongmin Fu, Daqing Sun

**Affiliations:** Department of Pediatric Surgery, Tianjin Medical University General Hospital, Tianjin, China

**Keywords:** circDOCK1, microarray analysis, biomarker, colorectal cancer, potential function

## Abstract

**Introduction:**

Endoscopic tissue biopsy combined with histopathology is the gold standard for the diagnosis of colorectal cancer (CRC); however, the invasive nature of this procedure hinders its acceptance by patients. Therefore, there exists a critical need to identify novel markers facilitating early CRC detection and prognosis. Circular RNAs (circRNAs) hold promise as novel clinical diagnostic markers. This study aimed to investigate the impact of circDOCK1 on CRC metastasis and prognosis as well as its underlying molecular mechanisms.

**Methods:**

We explored circRNA expression profiles in four pairs of CRC tissues and adjacent non-carcinoma tissues via microarray analysis. After Gene Ontology (GO), Kyoto Encyclopedia of Genes and Genomes (KEGG), and circRNA–miRNA network analyses, circDOCK1 was chosen for further investigation. We evaluated its clinical relevance in 80 CRC tissue pairs and adjacent controls, correlating circDOCK1 expression with clinical characteristics. Follow-up data from patient telephone interviews were analyzed for survival outcomes. Transfection efficiency was confirmed via qRT-PCR in HCT116 and SW480 colon cells, and the effects of circDOCK1 on cell proliferation, migration, and invasion were assessed.

**Results:**

Microarray data revealed 149 significantly differentially expressed circRNAs, including 71 upregulated and 78 downregulated circRNAs, in CRC tissues. CircDOCK1 exhibited elevated expression in patients with CRC and emerged as an independent prognostic factor. Kaplan–Meier curve analysis suggested that circDOCK1 expression is an unfavorable prognostic factor in patients with CRC. *In vivo* experiments revealed that circDOCK1 overexpression enhanced the proliferation, migration, and invasion of CRC cells, with consistent results upon circDOCK1 downregulation.

**Conclusion:**

These data indicate that circDOCK1 may play a role in promoting the proliferation, migration, and invasion of CRC cells, suggesting its potential as a CRC biomarker.

## 1 Introduction

Colorectal cancer (CRC) is a common malignant gastrointestinal cancer worldwide. In 2023, global projections anticipated 153,020 new CRC cases, resulting in an estimated 52,550 deaths (Siegel et al., 2023). The incidence rate of CRC in China is roughly equivalent to the global rate of approximately 20.2 cases per 100,000 people, making it the fifth most common type of cancer in the country (Zheng et al., 2024). In most patients, CRC is diagnosed at an advanced stage, with poor clinical treatment results and prognosis (Miller et al., 2022). Currently, CRC examinations mainly include physical examination, imaging, colonoscopy, and tumor biochemical assessments (Sun et al., 2018; Thomsen et al., 2018; Zhang et al., 2018; Burz et al., 2019). Although computed tomography is non-invasive, it is limited to morphological imaging and may misdiagnose residual stools as tumors, resulting in false positive results (Zhang et al., 2018). The blood-derived biomarkers carcinoembryonic antigen (CEA) and carbohydrate antigen 19-9 (CA19-9) are associated with CRC diagnosis and prognosis (Sun et al., 2018; Thomsen et al., 2018). However, their specificity diminishes in other malignancies such as ovarian and pancreatic cancers, as well as in benign conditions like inflammatory bowel disease, where CEA levels can also rise. Endoscopic tissue biopsy combined with histopathology is the gold standard for the diagnosis of CRC. However, it is not easily accepted by most patients due to the invasive nature of the examination (Burz et al., 2019). Therefore, there exists a critical need to identify novel markers facilitating early CRC detection and prognosis.

With the development of second-generation sequencing and bioinformatics technologies, research and understanding of circular RNA (circRNA) has become increasingly in-depth. CircRNA, characterized by its closed circular structure devoid of a 5′-end cap and a 3′-end polyadenylate tail, exhibits remarkable stability against exonucleases (Connelly et al., 2016). Notably, circRNAs hold promise as novel clinical diagnostic markers due to their ubiquitous presence, tissue development stage specificity, and evolutionary conservation (Li et al., 2015; Hsiao et al., 2017). Increasing evidence shows that circRNAs, as competitive endogenous RNA, participate in miRNA-binding competition in the cytoplasm, thus playing an important role in the carcinogenic process (Liang et al., 2020).

In this context, the present study conducted a high-throughput sequencing analysis of fresh CRC tissues alongside adjacent tissues from patients with CRC to identify differentially expressed circDOCK1. This investigation aimed to elucidate the expression levels of circDOCK1 and its association with relevant clinical data. Further exploration into the mechanistic involvement of circDOCK1 in CRC pathogenesis is warranted. By doing so, this study endeavors to furnish a foundational understanding of CRC etiology and to unveil potential new clinical markers.

## 2 Materials and methods

### 2.1 Patient selection

Demographic information, including sex, age, and body mass index, was collected from 80 patients at Tianjin Medical University General Hospital. Diagnosis of CRC was established through colonoscopy before surgery, preceded by stringent preoperative protocols, which included an 8-h water fast and a 24-h food restriction. The inclusion criteria encompassed patients with a pathological diagnosis of CRC who had undergone surgery, while the exclusion criteria were patients with TNM stage 0 or those with unknown staging. None of the patients presented contraindications to surgery or had previously undergone radical CRC procedures, adhering to the National Comprehensive Cancer Network (NCCN) guidelines. Following surgical resection, pathological assessment of the CRC tissue was conducted, accompanied by immediate preservation of portions of both the tumor and adjacent healthy tissues in liquid nitrogen for subsequent high-throughput whole-transcriptome sequencing. This study was approved by the ethics committee of Tianjin Medical University General Hospital (approval No. IRB2020-WZ-203). Written informed consent was obtained from all participants for the publication of this study.

### 2.2 Clinicopathological characteristics, prognosis analysis, and experimental groups

Clinicopathological attributes were documented for each patient, encompassing TNM stage, tumor diameter, degree of differentiation, presence of positive lymph nodes, and T stage. Outpatient visits or telephone follow-ups were used for evaluation. The experiments were performed using eight samples from four patients with CRC, including four CRC tissue samples and four adjacent healthy tissue samples. The tissues were divided into two groups: cancer and control.

### 2.3 circRNA sequencing and RNA library construction

Total RNA was isolated from CRC samples using TRIzol reagent according to the manufacturer’s instructions. RNA quantification and quality were assessed using a NanoDrop ND-1000. Evaluation of RNA integrity and absence of DNA contamination was achieved through denaturing agarose gel electrophoresis, where complete absence of DNA and absence of RNA degradation were confirmed. RNA integrity was deemed satisfactory with an RNA Integrity Number (RIN) ≥ 7. This was assessed using an Agilent 2,100 instrument. Removal of ribosomal RNA (rRNA) was executed using the Ribo-Zero rRNA Removal Kit (Illumina, United States) from the total RNA. The resulting rRNA-depleted RNA was then utilized for library construction employing the TruSeq Stranded Total RNA Library Prep Kit (Illumina, United States), following the manufacturer’s guidelines. Quality and quantity assessments of the constructed libraries were conducted using a BioAnalyzer 2,100 system (Agilent Technologies, United States). Libraries (10 p.m.) were denatured into single-stranded DNA molecules, captured on Illumina flow cells, amplified *in situ* as clusters, and sequenced for 150 cycles using an Illumina HiSeq Sequencer, according to the manufacturer’s instructions.

#### 2.3.1 circRNA sequencing analysis

High-throughput whole-transcriptome sequencing and subsequent bioinformatic analyses were performed. Briefly, paired-end reads were obtained using an Illumina HiSeq 4,000 sequencer. Following 3′ adaptor-trimming and removal of low-quality reads via the cutadapt software (v1.9.3), high-quality reads were aligned to the reference genome/transcriptome using STAR software. CircRNAs were detected and identified using DCC software40 and annotated using the circBase database. Raw junction reads for all samples were normalized to the total number of mapped reads and log2 transformed. Differential expression analysis of circRNAs was conducted utilizing t-tests, evaluating fold changes between the two groups. To account for multiple hypothesis testing, P-values were corrected using the Benjamini–Hochberg (BH) method. CircRNAs exhibiting fold change (FC) ≥ 2.0 with P-values ≤ 0.05 were classified as significant. To determine the associated mRNA (the circRNA-associated gene), coordinates were obtained from the RefSeq database based on the back-splicing site coordinates of the circRNA, as per established methods. Subsequently, Gene Ontology (GO; http://www.geneontology.org/) and Kyoto Encyclopedia of Genes and Genomes (KEGG; http://www.genome.jp/kegg) analyses were performed. Statistical significance was set at P < 0.05. The top 10 significant pathways of the upregulated and downregulated circRNAs were chosen to construct the pathway relationship network, which was based on the interaction data from KEGG analysis. A pathway relationship network was constructed to identify the regulatory effects of these pathways. Genomic sequence corresponding to the exons of circRNA coordinates were spliced based on chain-specific complementarity. For non-exonic circRNAs, the genomic sequence between circRNA coordinates was directly obtained. Subsequently, predicted sequences of circRNAs were utilized to forecast circRNA–miRNA interactions using custom Arraystar iRNA target prediction software, incorporating TargetScan and miRanda algorithms. The miRNA-binding sites on the differentially expressed circRNAs and the top five putative target miRNAs for the differentially expressed circRNAs were identified. The circRNA–miRNA network was constructed using the Cytoscape software.

### 2.4 Quantitative reverse transcription-polymerase chain reaction analysis

To validate the accuracy of the circRNA-seq data and investigate the association between circDOCK1 and prognosis, quantitative real-time polymerase chain reaction (qRT-PCR) was employed as a validation technique. Six circRNAs including four upregulated circRNAs (circSKA3_005, circDOCK1, circABR_010, and circPLCE1_005) and two downregulated circRNAs (circPDE5A_002 and circATRNL1_004) were selected and validated. The target RNA and internal parameters of each sample were subjected to real-time PCR reactions on an Applied Biosystems 7,500 Fast Real-Time PCR System (version 2.0.5; Roche, Basel, Switzerland) using SYBR Green qPCR SMix (Roche, Basel, Switzerland). Total RNA was reverse transcribed to synthesize cDNA using the Prime Script RT Reagent Kit (Perfect Real-Time; TaKaRa, Osaka, Japan). The data were analyzed using the 2^−ΔΔCT^ method. For primer design, an outward-facing primer was typically generated targeting an exon sequence proximal to the back-splice site (within 150 nt upstream or downstream) of the circRNA. The PCR protocol comprised an initial denaturation step at 95°C for 10 min, followed by 40 cycles of amplification (95°C for 10 s, 60°C for 60 s for fluorescence collection). Following completion of the amplification reaction, a final melt curve analysis was performed with the following steps: 95°C for 10 s, 60°C for 60 s, and 95°C for 15 s. The temperature was slowly increased from 60°C to 99°C (automatic instrument, ramp rate 0.05°C/s) to establish the melting curve of the PCR product. The primers for the circRNAs and internal parameters of *GAPDH* are listed in Table 1.

**TABLE 1:** Primer sequences for circDOCK1.

Genes	Sequences of primer (5′–3′)
*circSKA3_005*	F : GAGGAATAATAAAAGTACACGAGCA
	R : ACTGCTTGCAACAGGAGGAT
*circDOCK1*	F : GAAATCGTCCACAGTGACCT
	R : CACAGTGTCTCCGATCTGRAAA
*circABR_010*	F : AGATCGTGGACAAGATCATGGG
	R : GAACCAGCTTCCTCATCTCCA
*circPLCE1_005*	F : GCTGCGGAAACAGTACGTCA
	R : CCTTCTGTGAGTCCTCTGAACC
*circPDE5A_002*	F : TCATAGGGAAGAGAGAAATGGTCA
	R : AGGGGCACTGTTATCTGCAC
*circATRNL1_004*	F : AATTGCGGCAGTCCAGATCA
	R : GGTTCTGTTAACCTTGCCAACT
*GAPDH*	F : ATGACATCAAGAAGGTGGTGAAGCAGG
	R : GCGTCAAAGGTGGAGGAGTGGGT
siRNA	TCA​CAC​AGC​ATG​CTT​TTT​ATT

### 2.5 Cell culture and transfection

Human CRC cell lines, HCT116 and SW480, were obtained from the National Infrastructure of Cell Line Resources. Cells were cultured with 1,640% and 10% fetal bovine serum in a humidified incubator containing 5% CO_2_ at 37°C. The circDOCK1 overexpression plasmid was designed and transfected into cells at ∼50 confluence to promote circDOCK1 expression using Lipofectamine RNAiMAX (Life Technologies, Grand Island, NY, United States). Additionally, two small interfering RNAs (siRNAs) against circDOCK1 were designed and transfected to inhibit circDOCK1 expression using ViaFect (Promega, Madison, WI, United States). The target sequences for circDOCK1 siRNAs were as follows: siRNA-1: 5′-TCA​CAC​AGC​ATG​CTT​TTT​ATT-3′; siRNA-2: 5′-GUG​AAG​CUU​UUU​AUA​ACU​ATT-3′. After 48 h, knockdown and overexpression of circDOCK1 were confirmed via qRT-PCR.

### 2.6 *In vitro* cell growth assay

*In vitro* cell growth assays were performed using the cell counting kit-8 (CCK8). Cells were seeded in 96-well plates and were incubated with 10% CCK8 solution at 37°C and 5% CO_2_ for 2 h. A cell proliferation curve was drawn immediately by measuring the absorption at 450 nm using a multifunctional enzyme marker.

### 2.7 Transwell assay

Cells transfected with the designated constructs were collected 48 h post-transfection and subjected to Transwell assays to evaluate cell invasion. In these assays, 5 × 10^4^ transfected cells were seeded into the upper chamber of a 24-well Transwell system with a membrane featuring 8 mm diameter pores, in serum-free 1,640 medium. The lower chamber was filled with 1,640 medium containing 10% fetal bovine serum, serving as a chemoattractant. Inserts were then incubated for 24 h at 37°C. The remaining cells were discarded from the upper layer of the insert by scrubbing with a sterile cotton swab, and cells that migrated to the lower surface of the filters were fixed in 4% paraformaldehyde for 10 min at 15°C. Invading cells were examined, counted, and photographed using a digital microscope at 15°C for 10 min.

### 2.8 Scratch wound assay

HCT116 and SW480 cells transfected with plasmids overexpressing circDOCK1 and circDOCK1 siRNA were seeded in 24-well plates at 3 × 10^5^ cells/well. Two compound holes were created in each hole. Once the cells achieved 100% confluence, the culture plate was scratched along its central axis using a 10 µL pipette tip. The cells were then rinsed thrice with PBS to remove cell debris and floating cells. Afterwards, fresh medium was added, and the 24-well plates were returned to the incubator at a constant temperature of 37°C with 5% CO_2_. Wound healing within the scraped wound line was observed using an inverted light microscope at 0 and 24 h, and images of representative scrape lines were captured. Each duplicate well was examined, and each experiment was repeated in triplicate.

### 2.9 Statistical analysis

All statistical analyses were performed using SPSS version 24.0. Data are expressed as mean ± standard deviation. Student’s t-test was used to assess differences between two groups. Differences were considered statistically significant at P < 0.05.

## 3 Results

### 3.1 The screening process and the expression levels of circDOCK1 in CRC tissues and adjacent healthy tissues

**FIGURE 1:**
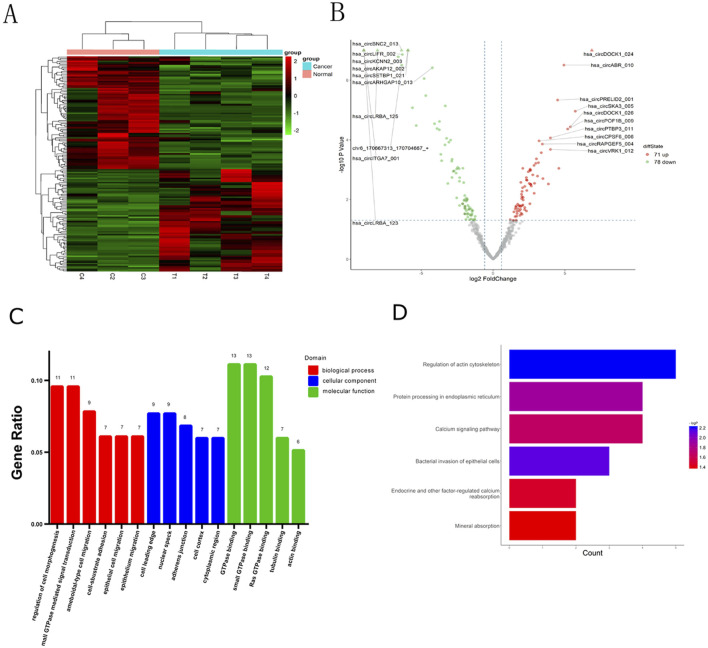
Identification of differentially expressed circRNAs in CRC and bioinformatics analysis. **(A)** CircRNA expression profiles among the CRC tissues and adjacent normal tissues. **(B)** Volcano plots of circRNAs differentially expressed. **(A)** (Heatmap) and **(B)** (Volcano Plot) were created using the ggplot2 package in R. **(C)** Gene Ontology (GO) includes three types of analyses: Biological Processes (red), Cellular Components (blue), and Molecular Functions (green). **(D)** Target mRNAs of the networks were functionally annotated using KOBAS prior to Kyoto Encyclopedia of Genes and Genomes (KEGG) pathway analysis.

The quality of RNA from these samples was assessed using an Agilent 2,100 Bioanalyzer, and the RNA concentration was measured using a Qubit 3 Fluorometer. Despite one adjacent healthy tissue yielding RNA of insufficient quality for sequencing, circRNAs from three adjacent healthy tissues and four CRC tissues were successfully sequenced. Analysis revealed 149 circRNAs exhibiting significant differential expression, meeting the criteria of FC ≥ 2.0 and P-values ≤ 0.05. Among these, 71 were upregulated and 78 were downregulated. A heat map and volcano plot of 149 differentially expressed circRNAs were constructed to illustrate the distinguishable circRNA expression profiles and variations in circRNA expression between the two groups (Figures 1A, B). Furthermore, the top 10 upregulated and downregulated circRNAs, ranked by FC in expression, are listed in Tables 2, 3, respectively. An FC threshold of 2.0 was utilized for inclusion.

**TABLE 2:** Top 10 upregulated circRNAs in CRC tissues screened by fold change (FC) and P-value.

circRNA	P-value	FC	logFC	chrom	strand	Gene
hsa_circDOCK1_024	2.99637E-08	116.9817631	6.870139828	chr10	+	DOCK1
hsa_circSKA3_005	1.09364E-05	52.62988468	5.717810329	chr13	—	SKA3
hsa_circDOCK1_026	3.64972E-05	41.43294311	5.372706398	chr10	+	DOCK1
hsa_circPOF1B_009	4.29682E-05	36.63141299	5.195009446	chrX	—	POF1B
hsa_circABR_010	3.11285E-07	30.47110636	4.929369978	chr17	—	ABR
hsa_circPLCE1_005	0.000895142	23.86245996	4.576670871	chr10	+	PLCE1
hsa_circPRELID2_001	4.58992E-06	22.40847467	4.485972544	chr5	—	PRELID2
hsa_circPTBP3_011	8.58386E-05	15.94295508	3.994847158	chr9	—	PTBP3
hsa_circVRK1_012	0.00020872	15.88707735	3.98978184	chr14	+	VRK1
hsa_circRAPGEF5_006	0.001978532	11.00832191	3.460522658	chr7	—	RAPGEF5

**TABLE 3:** Top 10 downregulated circRNAs in CRC tissues screened by fold change (FC) and P-value.

circRNA	P-value	FC	logFC	chrom	strand	Gene
hsa_circSCMH1_023	0.036665653	0.4220227	−1.244607494	chr1	—	SCMH1
hsa_circAFF1_005	0.045035	0.409147	−1.28931	chr4	+	AFF1
hsa_circRHOBTB3_020	0.049325	0.406399	−1.29903	chr5	+	RHOBTB3
hsa_circRBM23_004	0.04778	0.376269	−1.41016	chr14	—	RBM23
hsa_circLOC375190_005	0.023331	0.374606	−1.41656	chr2	+	FAM228B
hsa_circYY1AP1_008	0.029076	0.363421	−1.46029	chr1	—	YY1AP1
hsa_circFKBP3_003	0.024049	0.361519	−1.46786	chr14	—	FKBP3
hsa_circFAM190B_016	0.033637	0.357805	−1.48275	chr10	+	CCSER2
hsa_circHOOK3_003	0.044711	0.344406	−1.53782	chr8	+	HOOK3
hsa_circLPAR1_015	0.020387	0.343298	−1.54247	chr9	—	LPAR1

GO analysis was used to annotate and speculate on the functions of genes derived from these circRNAs (Figure 1C). GO analysis included three parts: biological process (BP), cell component (CC), and molecular function (MF). BP of GO analysis showed that the differentially expressed circRNAs were significantly related to the regulation of cell morphogenesis, small GTPase-mediated signal transduction, ameboidal-type cell migration, cell-substrate adhesion, epithelial cell migration, and epithelial migration. CC of GO analysis showed that differentially expressed circRNAs were significantly associated with the cell leading edge, nuclear speck, adherens junction, cell cortex, and cytoplasmic region. MF analysis revealed associations with small GTPase binding, GTPase binding, Ras GTPase binding, guanyl-nucleotide exchange factor activity, tubulin binding, and actin binding.

Based on the KEGG pathway analysis, we predicted the pathways affected by differences in circRNAs between CRC tissues and adjacent healthy tissues (Figure 1D). These pathways encompassed regulation of the actin cytoskeleton, bacterial invasion of epithelial cells, protein processing in the endoplasmic reticulum, calcium signaling, endocrine and other factor-regulated calcium signaling, and mineral absorption.

**FIGURE 2:**
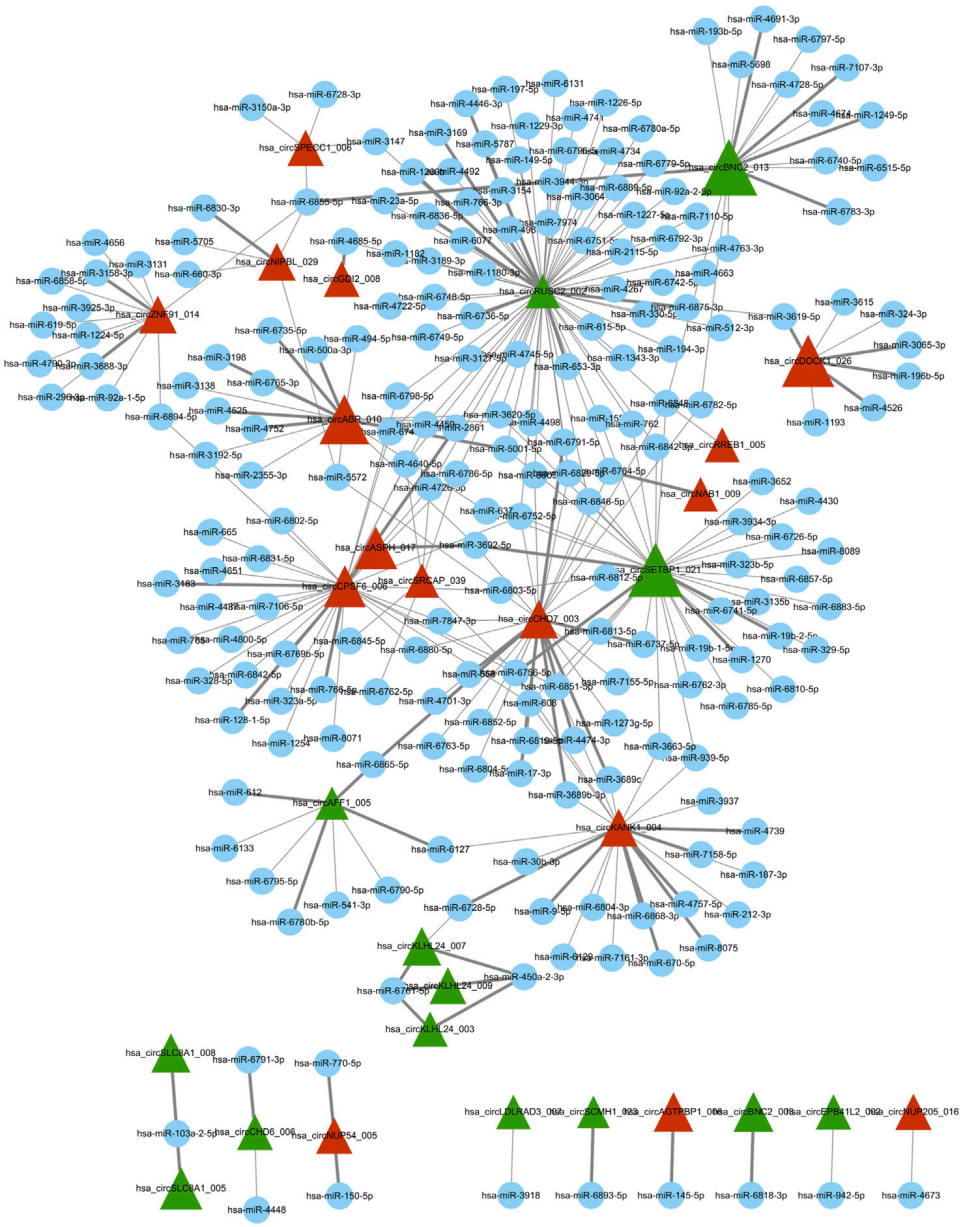
Differentially expressed circRNA/miRNA connection network. Red triangle represents upregulated circRNAs, green triangle represents downregulated circRNAs, and blue node represents miRNAs. In this figure was generated using the Cytoscape software.

Using miRNA target prediction software like miRanda, we constructed a circRNA–miRNA network, comprising 16 upregulated circRNAs and 14 downregulated circRNAs and their associated miRNAs (Figure 2). Notably, circBNC2, circRUSC2, circSETBP1, circABR, circCPSF6, and circDOCK1 exhibited a higher degree of regulation by multiple miRNAs compared to other circRNAs. Moreover, through GO and KEGG analysis, we found that circDOCK1 was associated with epithelial cell migration, regulation of cell morphogenesis, and regulation of actin cytoskeleton. These results suggest that circDOCK1 plays an important role in positively regulating cell migration.

**FIGURE 3:**
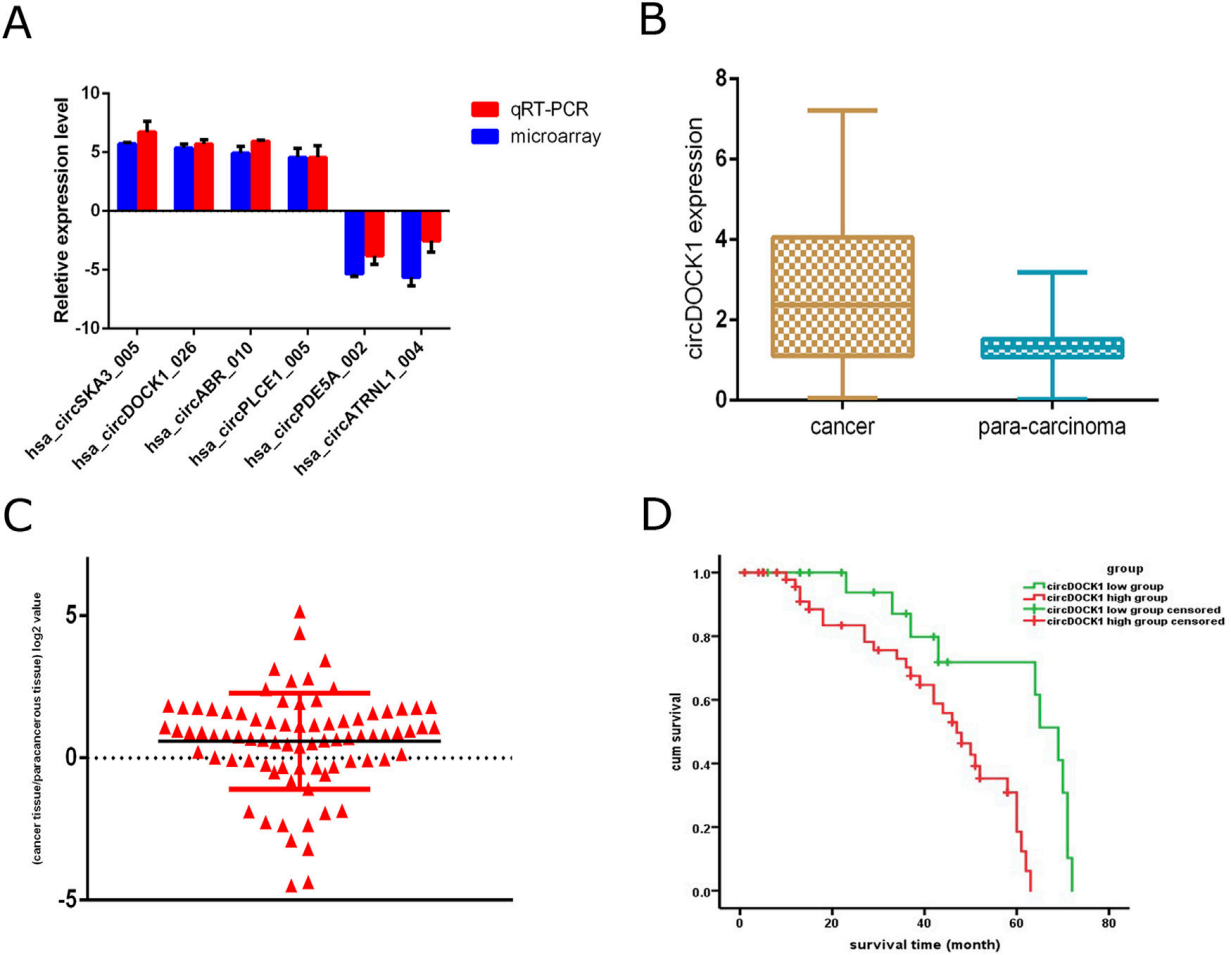
qRT-PCR validation of circDOCK1 expression levels and survival analyses. **(A)** qPCR analysis of circRNAs differentially expressed between different tissue types. **(B)** qPCR analysis of circDOCK1 expression in CRC tissues compared to paired adjacent healthy tissues (*n* = 80). **(C)** Logarithmic values of the ratios of circDOCK1 expression level. **(D)** Survival analysis of the two groups.

Six circRNAs were selected to confirm their differential expression between CRC tissues and adjacent healthy tissues via qRT-PCR. The results showed that four circRNAs (circSKA3_005, circDOCK1, circABR_010, and circPLCE1_005) were significantly upregulated in CRC tissues, whereas two circRNAs (circPDE5A_002 and circATRNL1_004) were significantly downregulated in CRC tissues. The results of the microarray analysis were consistent with those of the qRT-PCR (Figure 3A). CircDOCK1 was significantly overexpressed in CRC tissues. Consequently, circDOCK1 was selected for further studies because of its important role in the positive regulation of cell migration.

To assess the expression disparity of circDOCK1 between CRC tissues and adjacent healthy tissues, we conducted qRT-PCR analysis on 80 CRC tissue samples alongside their corresponding adjacent healthy tissues. Our findings indicate a notable elevation in the relative expression of circDOCK1 within CRC tissues compared to adjacent normal tissues (P < 0.01; Figure 3B). We categorized individuals into circDOCK1 low and high groups based on the logarithmic values of circDOCK1 expression ratios in CRC tissues relative to adjacent healthy tissues (where relative expression ratio > 1; logarithmic values > 0; Figure 3C).

### 3.2 circDOCK1 was a significant prognostic risk factor for patients with CRC

Comparison of circDOCK1 expression between the high- and low-expression groups showed a statistically significant correlation between circDOCK1 expression and tumor size, T stage, and number of positive lymph nodes. However, there were no significant differences in sex, age, BMI, differentiation, or distant metastases between the groups (Table 4).

**TABLE 4:** Relationship between the expression level of circDOCK1 and clinical pathology characteristics.

Characteristics	High expression group	Low expression group	c2/T value	P-value
Sex			0.016	0.898
Male	46	10		
Female	20	4		
Age	68.18 ± 8.445	67.42 ± 8.097	0.374	0.709
BMI	22.631 ± 2.258	22.44 ± 2.469	0.338	0.736
Tumor size	6.229 ± 2.514	4.825 ± 2.377	2.325	0.023
Differentiation			2.360	0.124
Secondary	20	13		
Poorly	36	11		
T stage			22.643	0.000
T1/T2	5	14		
T3/T4	51	10		
Positive lymph nodes	6.43 ± 3.08	1.50 ± 2.126	7.132	0.000
Distant metastasis			3.288	0.070
Yes	7	24		
No	49	0		

Spearman correlation analysis revealed a significant correlation between tumor size, T stage, number of positive lymph nodes, differentiation, and distant metastasis. There was a weak correlation between sex, age, and BMI between the groups (Table 5).

**TABLE 5:** Spearman correlation between the expression level of circDOCK1 and clinical pathology characteristics.

Characteristics	Spearman correlation	P-value
Sex	0.040	0.725
Age	0.016	0.886
BMI	0.020	0.862
Tumor size	0.222	0.048
Differentiation	0.293	0.008
T stage	0.462	0.000
Positive lymph nodes	0.681	0.000
Metastasis	0.392	0.000

Univariate analysis revealed that tumor size, number of positive lymph nodes, differentiation, distant metastasis, and expression level of circDOCK1 were prognostic factors. However, sex, age, BMI, and T stage were not prognostic factors. Multivariate analyses further demonstrated that tumor size, number of positive lymph nodes, differentiation, and circDOCK1 expression level were independent prognostic risk factors for patients with CRC. Thus, the circDOCK1 expression level in patients with CRC was used as an independent prognostic factor in the multivariate analysis (Table 6).

**TABLE 6:** Univariate and multivariate analyses of circDOCK1 expression.

	Univariate analysis			Multivariate analysis	
	P-value	SE	95% confidence interval	P-value	Relative risk
Sex	0.065	0.697 (0.377)	0.958–4.208		
Age	0.134	−0.027 (0.018)	0.939–1.008		
BMI	0.853	0.012 (0.066)	0.889–1.152		
Tumor size	0.038	0.129 (0.062)	1.007–1.285	0.044	1.195
Differentiation	0.002	1.022 (0.330)	1.456–5.299	0.006	3.186
T stage	0.141	0.517 (0.351)	0.843–3.337	0.104	0.386
Positive lymph nodes	0.000	0.160 (0.045)	1.075–1.282	0.004	1.234
Metastasis	0.000	−1.195 (0.233)	0.192–0.487	0.323	0.057
CircDOCK1 expression	0.001	0.293 (0.088)	1.128–1.594	0.046	1.418

We performed Kaplan–Meier curve analysis to identify whether circDOCK1 was related to the overall survival of patients with CRC. The follow-up period ranged from to 1–110 months, with a median of 78 months for all patients. The 5-year survival rate was 71.4%. Kaplan–Meier analysis revealed a significant difference in the overall survival rate between the high- and low-expression groups. The overall survival times were significantly longer in the low-expression group (65.04 ± 27.93 months) than in the high expression group (51.96 ± 23.878 months) (Figure 3D).

### 3.3 CircDOCK1 affected colon cell proliferation, migration, and invasion

**FIGURE 4:**
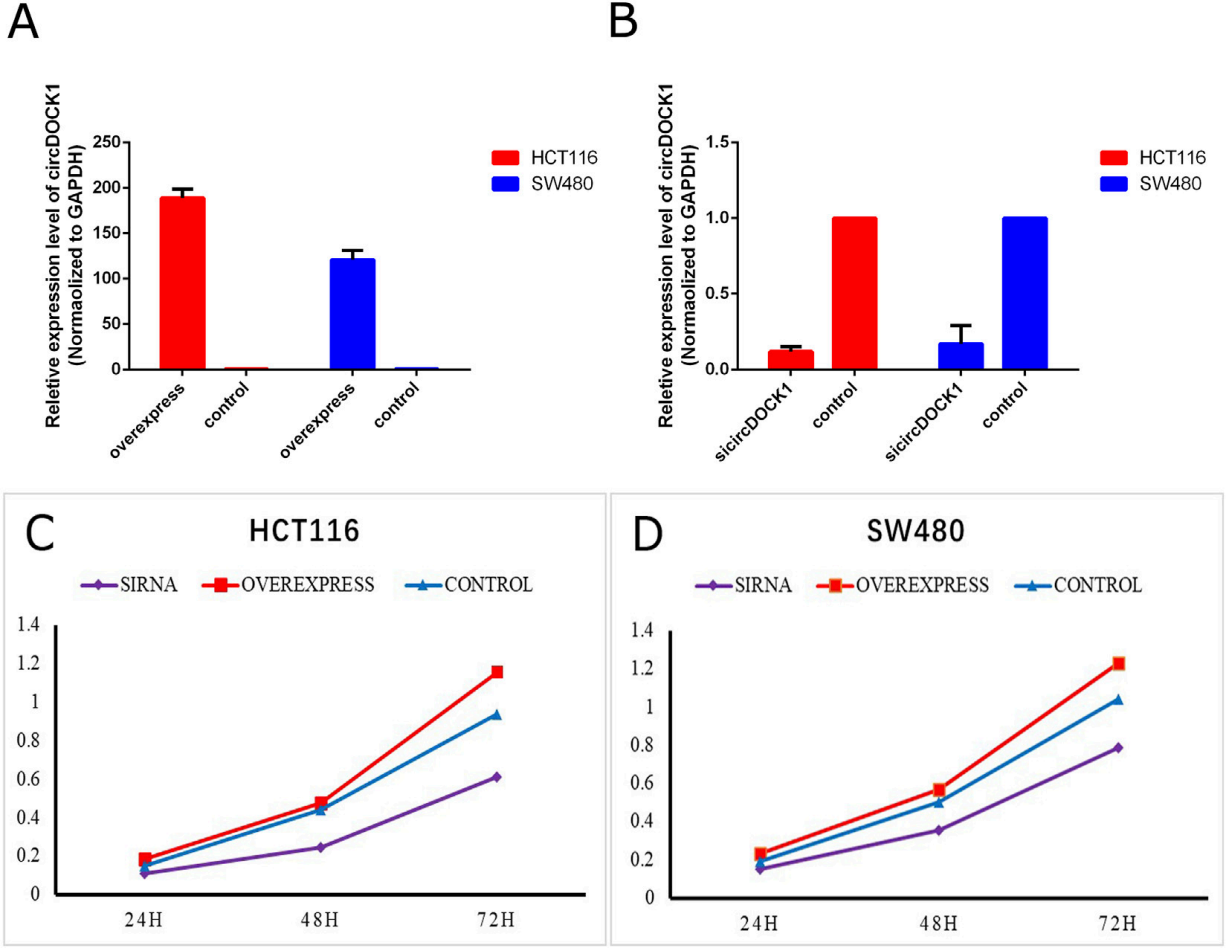
CircDOCK1 construction and proliferation capacity. **(A)** qPCR analysis of the overexpression efficiency of circDOCK1 following transfection with the respective plasmid in HCT116 and SW480 cell lines. **(B)** qPCR analysis of the knockdown efficiency of siRNA against circDOCK1 in HCT116 and SW480 cell lines. **(C, D)** CCK8 assay for the proliferation capacity of HCT116 and SW480 cell lines transfected with sicircDOCK1 and circDOCK1 plasmids.

**FIGURE 5:**
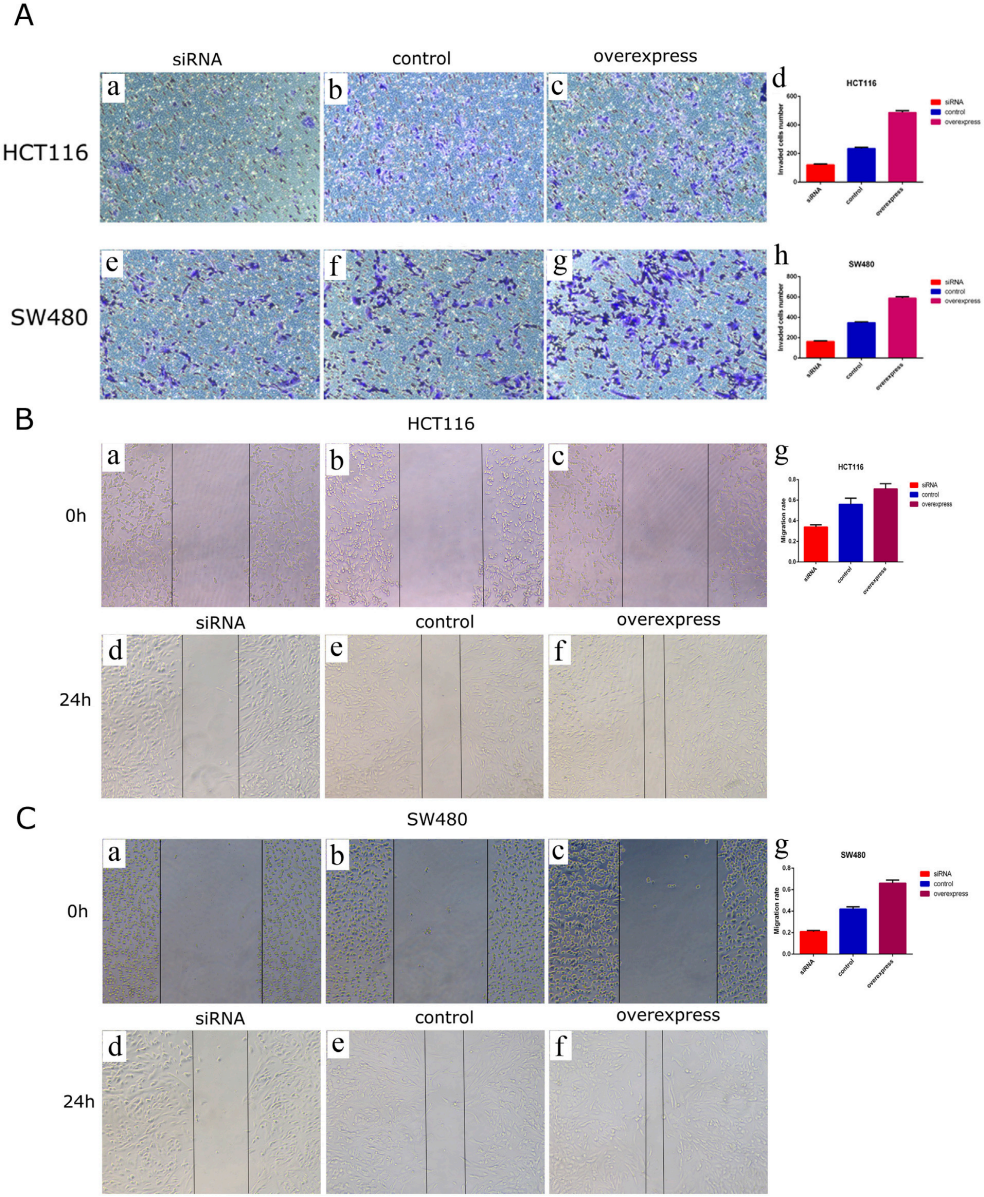
CircDOCK1 affects migration and invasion capacities in HCT116 and SW480 colon cells. **(A)** Transwell cell migration assays in HCT116 and SW480 colon cells. In **(A)**, some of the images are microscopic (a-c, e-g), while images d and h are bar charts depicting the application of comparison tests. **(B)** Scratch experiment in HCT116 colon cells. In **(B)**, some of the images are microscopic (a-f), while image g is a bar chart depicting the application of comparison tests. **(C)** Scratch experiment in SW480 colon cells. In **(C)**, some of the images are microscopic (a-f), while image g is a bar chart depicting the application of comparison tests.

To explore the biological function of circDOCK1 in CRC cells, an overexpression vector for the circDOCK1 plasmid and RNAi was constructed. According to circDOCK1 expression levels, colon cells were categorized into three groups: high, control, and low. Compared to the control group, qPCR revealed that the expression level of circDOCK1 in HCT116 and SW480 cells was significantly upregulated in the high group and significantly downregulated in the low group (Figures 4A, B). The results showed that the transfection was successful. The CCK8 assay indicated that after circDOCK1 was transfected into HCT116 and SW480 cells, the proliferation capacity of the cells in the high group with the overexpressed recombinant vector circDOCK1 was significantly enhanced compared with that in the control group. In the RNAi experiment, similar results were obtained for the control and low-dose groups (Figures 4C, D). These results suggest that circDOCK1 promotes the proliferation of HCT116 and SW480 cells. The scratch and Transwell cell migration assays revealed that after transfection of circDOCK1 into HCT116 and SW480 cells, cell migration and invasion capacities were remarkably elevated in the circDOCK1 overexpression group compared to the control group (Figure 5). The results obtained from the RNAi experiments aligned with those obtained from the overexpression experiments, indicating that circDOCK1 significantly affected the proliferation, migration, and invasion of HCT116 and SW480 colon cells.

## 4 Discussion

CRC is a prevalent malignancy of the digestive tract, marked by escalating morbidity and mortality rates globally (Tanne, 2020). Comprehensive approaches, including surgery, chemotherapy, radiotherapy, and biologically targeted therapy, are the major treatment strategies for CRC (Li J. et al., 2019). Unfortunately, late-stage diagnosis due to delayed symptom manifestation contributes to the dismal prognosis and substantial societal and economic burdens associated with this disease (Zerillo et al., 2017). Despite extensive efforts, the pathogenesis of CRC remains elusive, underscoring the imperative for in-depth exploration of its underlying mechanisms and the identification of novel biomarkers and pathways to enhance therapeutic interventions.

With the development of RNA sequencing, an increasing number of circRNAs have been discovered and identified in various cancer types (Xiao et al., 2020). As a special class of non-coding RNA characterized by a closed loop structure, circRNAs regulate tumor cell proliferation, apoptosis, and migration by controlling different genes and signaling pathways (Guo et al., 2023; Li et al., 2024). CircRNAs offer intriguing potential in cancer research. However, the oncological value of circRNAs in clinical settings remains unclear. Current research findings indicate that circRNAs can modulate the sensitivity of chemotherapy drugs in some tumors, such as in osteosarcoma, where they can promote progression and regulate cisplatin sensitivity through the miR-339-3p/IGF1R axis (Li et al., 2021). Additionally, it has been discovered that circRNAs play an important role in skin malignancies, especially in the context of skin tumor metastasis, and that circRNA in conjunction with nanomaterials may help to identify new therapeutic targets (Liu and Li, 2022; Xu and Li, 2023). CircRNAs have been reported to exert diverse functions, acting as miRNA sponges, binding partners for RNA binding proteins, traps for mRNAs, and modulators of transcription, and in some instances, encoding proteins directly (Yu and Kuo, 2019). In addition, circRNAs exhibit stronger structural stability, tissue specificity, and expression specificity than linear RNA (Du et al., 2024). Consequently, their distinct attributes render circRNAs promising candidates as biomarkers for early diagnosis and prognostic assessment in CRC and other malignancies (Li Y. F. et al., 2020; Liu and Li, 2024). Thus, elucidating the mechanisms governing circRNA expression and function holds not only theoretical significance, but also considerable clinical relevance for CRC research.

In this study, we randomly selected four CRC samples and their para-cancerous counterparts for high-throughput whole-transcriptome sequencing. Unfortunately, one CRC sample was excluded due to inadequate RNA quantity and quality, likely attributable to prolonged transportation time. We further obtained the expression profile of circRNAs from these samples and performed subsequent bioinformatics analysis. We identified 149 differentially expressed circRNAs, including 71 upregulated and 78 downregulated circRNAs in CRC. The dysregulated expression of numerous circRNAs in CRC tissues compared to matched normal tissues suggests their potential involvement in CRC pathogenesis. CircRNA is correlated with the occurrence of tumors, development, proliferation, invasion, and metastasis (Artemaki et al., 2020). For example, circRNA_104916 is associated with cell proliferation, migration, and epithelial–mesenchymal transition in CRC (Min et al., 2019). Additionally, circRNA_0055625, identified as being upregulated, has been linked to tumor growth, metastasis, and pathological staging in CRC [20]. Moreover, it potentially functions as a miR-106b sponge to modulate CRC biology. Numerous circRNAs are associated with tumorigenesis, development, and metastasis in CRC, including circ_0060745, circ_101951, circ_102958, circ_0004585, circ_103809, circ_100290, and circ_0026344 (Bian et al., 2018; Min et al., 2019; Li J. et al., 2020). Our results are consistent with those of previous studies. However, the precise underlying mechanisms by which circRNAs contribute to the tumorigenesis, development, and metastasis of CRC remain unclear and require further investigation.

To gain insights into the potential functions of the differentially expressed circRNAs, GO and KEGG pathway analyses were performed. CircRNA–miRNA predictive interaction networks were also established to predict the relationships between circRNAs and miRNAs. We found that one upregulated circRNA, circDOCK1, may play an important role in the positive regulation of cell migration in CRC. We compared circDOCK1 expression in 80 pairs of tissues from patients with CRC using qPCR. Our findings revealed a significant upregulation of circDOCK1 in CRC tissues compared to matched adjacent non-tumor tissues. Furthermore, circDOCK1 expression was associated with tumor size, T stage, and the number of positive lymph nodes. Multivariate analyses identified circDOCK1 expression as an independent prognostic factor for CRC. Additionally, Kaplan–Meier analysis unveiled a correlation between circDOCK1 expression and overall survival in patients with CRC, suggesting its potential utility as a prognostic biomarker for predicting outcomes in CRC. Furthermore, our study uncovered a correlation between altered circDOCK1 expression and key cellular processes including proliferation, apoptosis, invasion, and migration in HCT116 and SW480 cells. DOCK1, an important regulator of cell progression, is associated with metastatic processes in human cancer and accelerates cancer progression (Chiang et al., 2019). CircDOCK1, derived from mRNA DOCK1, exhibits varying roles in tumor initiation and progression across distinct cancer types (Cui and Xue, 2020). Liu et al. demonstrated that circDOCK1 expression was upregulated in bladder cancer tissues. However, circDOCK1 knockdown significantly suppressed bladder cancer cell proliferation, migration, and invasion by interfering with the circDOCK1/hsa-miR-132-3p/Sox5 pathway (Liu et al., 2019). Moreover, circDOCK1, functioning as a competing endogenous RNA (ceRNA), modulates BIRC3 expression, prompting apoptosis in oral cancer (Artemaki et al., 2020; Wang et al., 2018). These studies suggest that circDOCK1 may function as a potential prognostic biomarker and therapeutic target in bladder and oral cancers. Similar to the functions of circDOCK1 affecting CRC proliferation, migration, and invasion that we identified, circDOCK1 can serve as a potential prognostic biomarker and therapeutic target for CRC, laying the foundation for subsequent drug development.

In conclusion, our study identified a suite of differentially expressed circRNAs in CRC tissues compared to healthy tissues, and subsequent GO and KEGG pathway analyses uncovered their pivotal biological roles. We further constructed a circRNA–miRNA interaction network to elucidate the underlying molecular mechanisms. Validation of circRNA expression in CRC tissues through qRT-PCR confirmed that circDOCK1, a prominent circRNA, is significantly associated with the clinicopathological characteristics of patients with CRC and serves as a prognostic risk factor. Moreover, circDOCK1 expression levels were found to influence the behavior of colon cells, indicative of its involvement in the progression of CRC. These findings underscore the potential of circDOCK1 as both a biomarker and a therapeutic target for the management of CRC. Despite these promising observations, the precise mechanisms underlying the influence of circDOCK1 on CRC pathogenesis remain elusive and warrant further investigation.

## Data Availability

The datasets presented in this study can be found in online repositories. The names of the repository/repositories and accession number(s) can be found below: https://www.ncbi.nlm.nih.gov/, PRJNA865763.
