# Supplementary material for: Circular RNA microarray expression profile and potential function of circDOCK1 in colorectal cancer
Source: Front Genet. 2025 Feb 4;16:1443876. doi: 10.3389/fgene.2025.1443876 (PMC11832710; doi:10.3389/fgene.2025.1443876)
Supplement: Supplementary file 1 [file DataSheet1.pdf]

Authorization of The Ethical Committee of Tianjin Medical University General Hospital

Ethical NO. IRB2020-WZ-203

Article title: **Circular RNA microarray expression profile and potential function of circDOCK1 in colon cancer**		
Corresponding author: Daqing Sun	First Authors: Guojing Zhang	Application departments: Department of Pediatric Surgery
Assume unit: Tianjin Medical University General Hospital	Application date: December, 2020	

Abstract:

Abstract:

Background: Aberrant expression of circular RNA (circRNA), a novel class of endogenous noncoding RNAs, contributes to a variety of diseases, including several types of cancers. In this study, we analyzed the effect of circDOCK1 on the metastasis and prognosis of colon cancer and explored its possible molecular mechanism.

Method: To verify the effect, we explored the circRNA expression profiles in 4 pairs of colon cancer tissues and 3 adjacent non-carcinoma tissues via microarray analysis. After the GO, KEGG analysis, and circRNA-miRNA network, circDOCK1 was selected for further study and the potential clinical value of circDOCK1 were investigated in 80 pairs of colon cancer tissues and adjacent normal controls. Moreover, the clinical and pathology data of the colon cancer patients were collected to investigate the relationship between the expression level of circDOCK1 and clinical characteristics. In addition, to explore the impact of its expression on survival outcome, the patient was followed up by telephone. HCT116 and SW480 colon cells were transfected, and the transfection efficiency was verified by qRT-PCR. The effects of circDOCK1 on the proliferation, migration, and invasion of colon cancer cells were detected by CCK8, Transwell migration, and scratch wound assays.

Results: The microarray data showed 149 significantly differentially expressed circRNAs including 71 upregulated and 78 downregulated in the colon cancer tissues. CircDOCK1 was highly expressed in colon cancer patients. Further analysis revealed that circDOCK1 was an independent prognostic factor. Kaplan-Meier curve analysis suggested that circDOCK1 expression is an unfavorable prognostic factor in colon cancer patients. The vivo experiments results revealed that overexpression of circDOCK1 enhanced the proliferation, migration, and invasion of colon cancer cells. The same result can be obtained after lowing circDOCK1.

Conclusion: These data indicate that circDOCK1 might play a role in promoting proliferation, migration, and invasion of colon cancer cells and be a potential biomarker of colon cancer.

Comments of Examination: After examination, " **Circular RNA microarray expression profile and potential function of circDOCK1 in colon cancer**", conform "International ethical guidelines for biomedical research involving human subjects (2002)" developed by Council For International Organizations Of Medical Sciences (CIOMS) in collaboration with World Health Organization (WHO), researchs in this article are approved.

Ethical Committee
Tianjin Medical University General Hospital
Tianjin,China
December, 2020
